# Specific approaches and limitations in (multi)-omic Mendelian randomization

**DOI:** 10.1016/j.jlr.2024.100619

**Published:** 2024-08-13

**Authors:** Arjen J. Cupido, Mingqi Zhou, Aldons J. Lusis, Marcus Seldin

**Affiliations:** 1 Department of Internal Medicine, Tergooi MC, Hilversum, The Netherlands; 2 Department of Biological Chemistry and Center for Epigenetics and Metabolism, University of California, Irvine, California, USA; 3 Departments of Human Genetics, Medicine, and Microbiology, Immunology, & Molecular Genetics, David Geffen School of Medicine of UCLA, Los Angeles, California, USA

Mendelian Randomization (MR) is a statistical method aiming to assess the causality of an exposure X on an outcome Y, using data from Genome-Wide Association Studies (GWAS) (1). An “exposure” could, for example, include a clinical risk factor, plasma metabolite or protein, or a gut bacterial species, while an “outcome” could include a disease or any complex trait. MR studies aim to provide an additional layer of evidence for a particular causality hypothesis over and above typical association studies and can be conducted with both individual-participant data as well as with summary-level GWAS data. Data from GWAS studies are readily available and conducting MR studies is much less expensive than experimental studies in either animal models or in human trials. As a result, MR has been a widely used and powerful tool in suggesting causal interactions between outcomes. For example, over a decade ago MR was used to show that circulating high-density lipoprotein (HDL) levels, despite being highly correlated with cardiovascular disease (CVD), are not causally associated with CVD, thus shifting a focus in our understanding of how lipids drive increases atherosclerotic risk (2).

Nevertheless, MR studies have limitations in terms of both methodology and interpretation, and caution is warranted in putting too much weight on the findings. In particular, with the rise of high throughput data such as microbiomics, proteomics, metabolomics, and RNA-Seq available in large population datasets, potential confounding issues such as pleiotropy and multiple comparisons become increasingly important to consider.

The editors of the *Journal of Lipid Research* felt that a critical review of MR applications would be timely given the large number of submissions employing the approach. For example, in 2022, 43 papers referenced on Pubmed could be found using the terms “mendelian randomization” and “microbiome”. This number increased 6-fold (267) in 2023 and by July of 2024 has surpassed to 353 publications. While many of the MR studies provided important results linking lipids or other molecules to clinical traits, other submissions failed to properly consider the limitations of the method. While not exhaustive, the goal of this commentary is to highlight considerations for MR usage and application with a particular focus on circulating molecules such as lipids and proteins.

## Fundamental MR Assumptions, Approaches and Limitations in Complex Systems

MR relies on three main assumptions: 1. The genetic variants used as instrumental variables are associated with the exposure, 2. The genetic variants are associated with the outcome only through the associated exposure, 3. The genetic variants are independent of other factors which affect the outcome. It is often difficult to meet all three assumptions because of unmeasured or unknown confounding, and several methods have been developed that relax one or more of these assumptions, though at the cost of power. Here, we discuss several commonly used MR approaches (Table 1) and specific pipelines (Table 2), but refer the reader to other papers providing more comprehensive summaries and discussions of assumptions and methods for MR investigations (3, 4, 5).

**Table 1:** Generalized MR approaches used

MR Approach	Description	Usage
One-Sample MR	Uses genetic- and metadata from individual participants for both exposure and outcome associations.	Ideal for investigating potential confounders and interactions, as well as non-linear effects.
Two-Sample MR	Uses summary statistics from two different samples for the exposure and outcome.	Ideal for leveraging existing large-scale summary data from genome-wide association studies (GWAS).
MVMR (Multivariable MR)	Extends MR to simultaneously estimate the causal effects of multiple exposures on an outcome.	Useful for disentangling the effects of correlated exposures and adjusting for confounders.
Reverse MR	Assesses the direction of causality by using the outcome as the exposure and vice versa.	Helps determine the direction of causality and identify bidirectional relationships.
Factorial MR	Assessing the combined effect of two + separate exposures on an outcome (by using individual-participant data).	Useful to explore the joint effect of two drug targets or risk factors.
Mediation MR	A framework to explore mediation of the effect of one exposure on an outcome by another factor.	Useful to explore the causal effect of multi-omic layers on an outome.

**Table 2:** Most commonly employed MR pipelines (3)

MR Method	Description	Pros	Cons	Sensitivity Checks
IVW (Inverse Variance Weighted)	Combines the effect estimates from multiple genetic variants, weighting them by the inverse of their variance.	- High statistical power - Handles multiple instruments - Simple implementation	- Sensitive to pleiotropy (where genetic variants affect multiple traits) - Assumes no measurement error in genetic variants	- MR-Egger regression - MR-PRESSO - Leave-one-out analysis
MR-Egger Regression	Similar to IVW but allows for a non-zero intercept, accounting for pleiotropic effects.	- Controls for pleiotropy - Provides an estimate of pleiotropy effect	- Lower statistical power compared to IVW - Assumes that pleiotropy is uncorrelated with the genetic variant-outcome association	- Testing the intercept - Heterogeneity tests
Weighted Median	Uses the median of the weighted instrumental variable estimates, providing a consistent estimate even if up to 50% of the instruments are invalid.	- Robust to invalid instruments - Simple interpretation	- Less efficient than IVW - Requires a large number of instruments for accuracy	- Comparison with IVW and MR-Egger - Simulation analyses
MR-PRESSO	Identifies and corrects for horizontal pleiotropic outliers, providing a more robust estimate.	- Detects and corrects for outliers - Provides global test for pleiotropy	- Computationally intensive - Requires a large sample size for outlier detection	- Outlier tests - Global test for pleiotropy

Designing MR studies requires careful consideration of the potential limitations of the method. A list of selected limitations that can confound MR studies (and especially multi-omic MR studies) include.

1 *Pleiotropy*: Genetic variants may have effects on multiple traits (pleiotropy), influencing the outcome through pathways other than just one specific trait, such as one metabolite. Therefore, the analysis could give misleading results.
2 *Population Stratification*: Differences in allele frequencies across populations due to ancestral background can confound MR results if not properly accounted for, leading to spurious associations. For this reason, replications of results across multiple independent cohorts could increase reliability of the results
3 *Weak Instrument Bias*: If the genetic variants used as instruments are weakly associated with “omics” measures, the MR estimates can be biased and imprecise, leaning towards the null effect.
4 *Measurement Errors*: Inaccurate or imprecise measurement of variables such as microbiome composition and metabolite levels can lead to biased estimates of the causal effect, if any exists.
5 *Reverse Causation*: Even though MR is designed to mitigate reverse causation, complex bidirectional relationships might still complicate the interpretation. This is particularly relevant for systems such as interactions between host genetics, microbiome composition, environmental factors and resulting molecular measures being assayed.
6 *Confounding factors*: Unaccounted confounding factors may still influence the results of MR associations. The regulatory networks determining commonly used molecular measures (ex. metabolomics in plasma) can be highly complex and dynamically regulated from multiple organs. Thus, factors driving strong associations such as tissue-specific pathways could be missed and lead to false characterization of causality in MR for downstream measures which also associate.
7. *Limited Generalizability*: Findings from specific populations or datasets may not be generalizable to other populations, particularly if there are differences in genetic architecture, environmental exposures, or lifestyle factors.

## Specific Approaches to Address Limitations in (Multi-omic) Mendelian Randomization Studies

### Consideration of biological pathways

When conducting an MR investigation, it is important to have a clear research question with well-defined exposure and outcome variables, as well as consideration of the biological relevance of these exposures and outcomes (6). An example where careful investigation of the exposure resulted in the avoidance of spurious results is the effect of Interleukin-6 (IL-6) signaling on the risk for CVD (7). Physiologically, inhibition of IL-6 signaling can be achieved by either lowering IL-6 levels or by blockage of the IL-6 receptor (IL-6R). Interestingly, inhibition of IL-6 signaling by variants within the *IL6R* (encoding the IL-6R) region results in lower CRP levels, but increased IL-6 levels. Thus, to avoid invalid results, researchers wishing to investigate the effect of inhibition of IL-6 signaling by using a GWAS of IL-6 levels for their exposure variable should exclude the *IL6R* region from their analyses. Similarly, researchers interested in dissecting age-related phenotypes in a multiethnic cohort initially applied genome-wide association mapping of a multi-trait principle component to narrow their candidate list to 27 genomic loci (8). Mendelian randomization was then applied to this select list of loci to infer causal mechanisms, enabling the authors to conclude that the interaction between vascular cell adhesion molecule 1 and apolipoprotein(a) presented a high-confidence candidate set to study for further analyses. In general, these studies utilize prior knowledge and/or additional analytical methods to limit the amount of pleiotropy among instrumental variables and exposures.

### Consideration of measurement techniques

The biological relevance mentioned above also extends to the measurement method used for the exposure/outcome variable. For example, MR papers using 16S genomic data on the microbiome should be extremely careful in claiming causality, as 16S sequencing lacks the depth to reliably quantify bacteria all the way down to the species level and thus often relies on imputation or use of higher taxonomic classes. Similarly, GWAS in lipidomics often employ lipidomic techniques that are unable to separate isomers. These measurement limitations can impair the analysis by increasing risk for pleiotropy or invalidating basic MR assumptions. Researchers wishing to conduct analyses on the microbiome or lipidome should carefully investigate the methods of the derived GWAS data before conducting MR analysis.

### Statistical and methodological approaches

A variety of methodological approaches have also been developed to address the limitations listed earlier, of which many are listed in table 2. It is important to carefully address the results of all of the included main and sensitivity methods and a different set of sensitivity methods might be required for each analysis. Other options for sensitivity analysis include using positive or negative controls, colocalization, or subgroup analyses (3). For example, a recent study adopted a “contamination mixture” approach to focus on reducing the influence of pleiotropy on results, where the authors were able to further parse apart mechanisms of lipoprotein associations to cardiovascular disease (9). Thoughtful data aggregation methods additionally help limit the number of comparisons and create a more biologically informed set of comparisons to test using MR. In addition, with the rise of multi-omic GWAS studies, suggestions have been made to adopt approaches that encompass network modeling either in parallel with or instead of MR to infer causation (10). A large number of network modeling approaches have been developed, each with different strengths/constraints. Broadly, these applications enable a global, typically graph-based, view of multiple variables, offering appeal in capturing larger numbers of interacting variables. Integration of MR and network models can present challenges in terms of how modules are assigned/constructed and which variables are used as instruments, but scalable approaches are being developed to enhance the combined utilization of these methods (11, 12).

### Integration with experimental results

The most definitive method of validation is through experimental validation. Experimental models such as mice and cultured cells offer the ability to fix genetic background and environmental variables, enabling researchers to definitively demonstrate that observations could be attributed to a single variable (for example, single gene perturbation). With the development of high-throughput approaches to assay causal interactions experimentally, integration with MR associations presents an intriguing path forward. Systematic integration of experimental model results with MR associations will likely be an important approach to narrow the mechanisms involved to candidate causal pathways.

## Conclusion

Mendelian randomization is a powerful, relatively novel tool in biomedical research. While MR holds much promise due to the availability of public data, it is very important that researchers carefully design their studies and where possible add additional layers of evidence that can increase the validity of their findings. These considerations become increasingly relevant when applying to multi-omic data with large numbers of measurements accompanied by technical and biological considerations. This review highlighted some of the limitations and pitfalls, and we would like to encourage the submission of work that adheres to these guidelines for consideration in the Journal of Lipid Research.
